# Metachronous Primary Pancreatic Neuroendocrine Tumor and Adenocarcinoma: A Case Report

**DOI:** 10.1002/cnr2.70595

**Published:** 2026-06-04

**Authors:** Anastasia S. Fatyanova, Maria E. Kalinina, Dina F. Islamova, Igor O. Shchekoturov, Julia V. Lerner, Nadezhda A. Stashevskaya, Pavel Burko, Ilias Miltiadis

**Affiliations:** ^1^ Sechenov University Moscow Russia; ^2^ RUDN University Moscow Russia; ^3^ Synergy University Moscow Russia; ^4^ Department of Biomedicine Neuroscience and Advanced Diagnostics (BIND), University of Palermo Palermo Italy

## Abstract

**Background:**

Pancreatic ductal adenocarcinoma (PDAC) has a dismal prognosis, with most patients presenting with advanced disease. Surgical resection remains the only potentially curative option, yet recurrence rates are high.

**Case:**

We present a rare case of metachronous primary pancreatic neuroendocrine tumor (PanNET) and PDAC in a 71‐year‐old female. Initial management of stage IA PanNET (pT1N0M0) involved pancreatoduodenectomy (Whipple procedure) in 2022. One year later, the patient developed primary PDAC (pT2N1M0, stage IIB) in the remnant pancreas and was treated with completion pancreatectomy. Postoperative complications (reactive thrombocytosis and type 3c diabetes) precluded adjuvant chemotherapy. Subsequent hepatic progression was controlled for 12 months with first‐line gemcitabine before progression necessitated second‐line irinotecan.

**Conclusion:**

This case highlights the challenges of managing sequential primary pancreatic malignancies, the impact of total pancreatectomy complications on therapeutic options, and the potential for disease control with sequential systemic therapy even after aggressive recurrence.

## 1 Introduction

Pancreatic cancer is projected to become the second leading cause of cancer‐related mortality worldwide by 2030 [1, 2]. Pancreatic ductal adenocarcinoma (PDAC) constitutes approximately 95% of cases and is frequently diagnosed at locally advanced or metastatic stages (> 80%), where systemic chemotherapy forms the cornerstone of management [3, 4, 5]. Surgical resection (partial or total pancreatectomy) offers the only potential for a cure in patients with localized disease, yet the 5‐year survival rate remains poor (ranging from 0% to 33% on the basis of tumor size and stage) [6, 7]. Outcomes are influenced by factors such as margin status (R0 vs. R1), lymph node involvement, vascular invasion, and patient performance status [6, 8]. Despite advances, recurrence rates postresection are high, underscoring the need for effective adjuvant and metastatic therapies [9]. The occurrence of metachronous primary pancreatic malignancies of differing histologies (e.g., neuroendocrine tumor followed by adenocarcinoma) is exceptionally rare and presents unique management challenges. Unlike synchronous “collision” tumors which occur simultaneously or within 6 months, metachronous tumors appear sequentially with an interval greater than 6 months. We report such a case with a 12‐month interval, detailing the clinical course, therapeutic interventions, and outcomes.

## 2 Case Report

### 2.1 Initial Presentation and Diagnosis of PanNET (2022)

**FIGURE 1:**
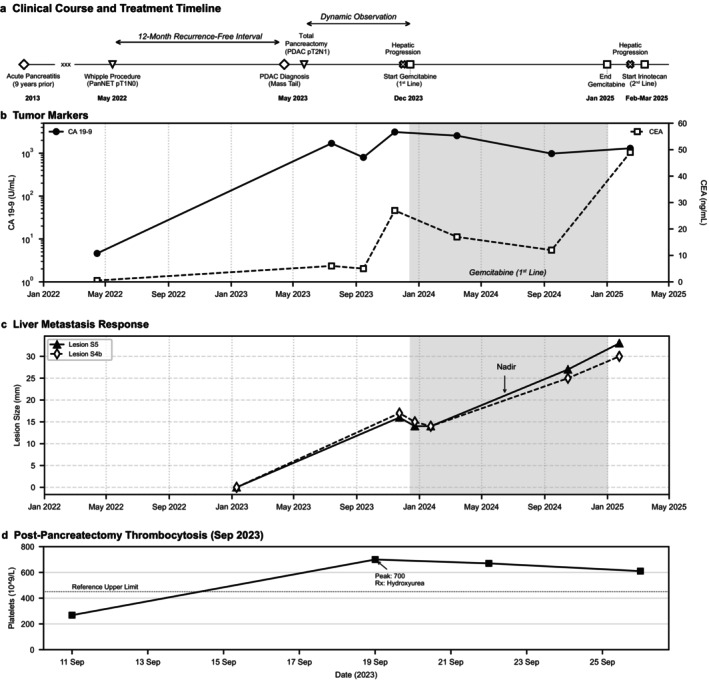
(a) Clinical course and treatment timeline, dynamics of (b) Tumor markers and (c) Liver metastasis lesion size, (d) Timeline of post‐pancreatectomy thrombosis.

**FIGURE 2:**
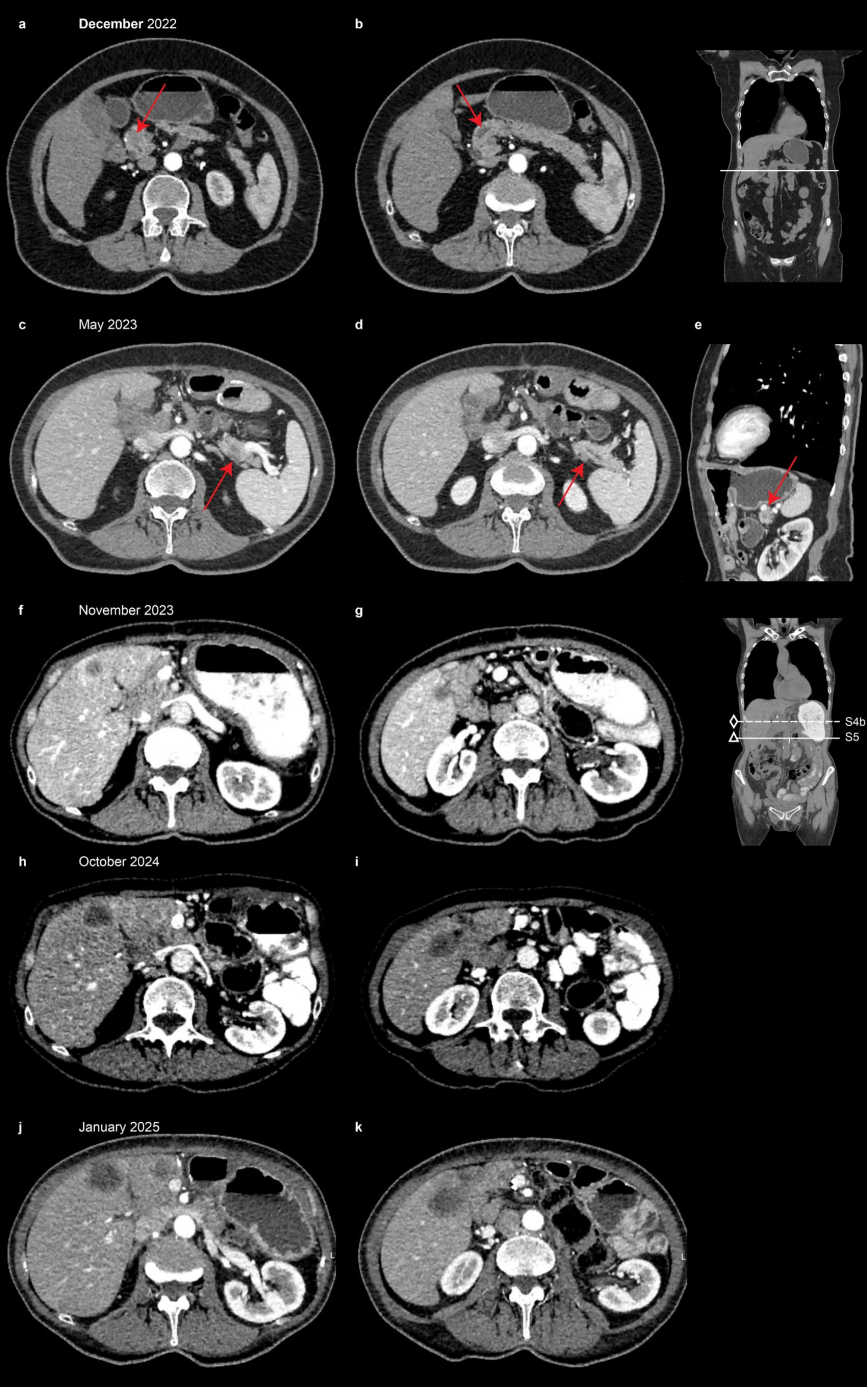
CECT of the abdomen (portal venous phase). December 2022: (a) Axial plane, demonstrating a hypovascular lesion measuring 18 × 13 mm within the pancreatic head. (b) At the level of this lesion, an abrupt interruption of the main pancreatic duct is observed, with proximal ductal dilatation up to 7 mm. No imaging evidence of extrapancreatic extension beyond the pancreatic parenchyma was identified. May 2023: (c) Hypovascular lesion with indistinct margins measuring 17 × 16 mm in the pancreatic tail, (d) Associated with infiltration of the surrounding peripancreatic fat and (e) Close contiguity with the splenic vein. November 2023: Hypovascular hepatic lesions. Two target lesions were selected for analysis: (f) One located in segment IVb measuring 17 mm and another (g) In segment V measuring 16 mm. October 2024: Hypovascular hepatic lesions. Two predefined target lesions exhibited significant interval growth: (h) A lesion in segment IVb increased in maximal diameter from 14 mm to 25 mm, whereas (i) The second lesion in segment V enlarged from 14 mm to 27 mm. January 2025: Hypovascular hepatic lesions. Two target lesions showed a marked increase in size: (j) One in segment IVb enlarged from 14 mm to 30 mm, and (k) The other in segment V increased from 14 mm to 33 mm.

A 71‐year‐old female presented with acute pancreatitis (resolved conservatively) that had occurred 9 years prior, and was treated conservatively at her local hospital (Figure 1a). In 2022, surveillance imaging revealed a 20 × 11 × 14 mm mass in the pancreatic head causing abrupt upstream pancreatic duct dilation (7 mm). The patient was admitted to Sechenov University Clinical Hospital. The levels of tumor markers (CEA and CA 19‐9) were within normal limits. Endoscopic ultrasound (EUS) revealed a 16 × 18 mm hypoechoic head mass with elastographic features suggestive of stiffness adjacent to but not invading the superior mesenteric vein. EUS‐guided fine‐needle biopsy (FNB) histopathology confirmed a well‐differentiated neuroendocrine tumor (Grade 1, Ki‐67 < 3%). Staging contrast‐enhanced computed tomography (CECT) (chest/abdomen/pelvis) revealed no evidence of metastatic disease (Figure 2a,b).

### 2.2 Treatment of PanNET

**FIGURE 3:**
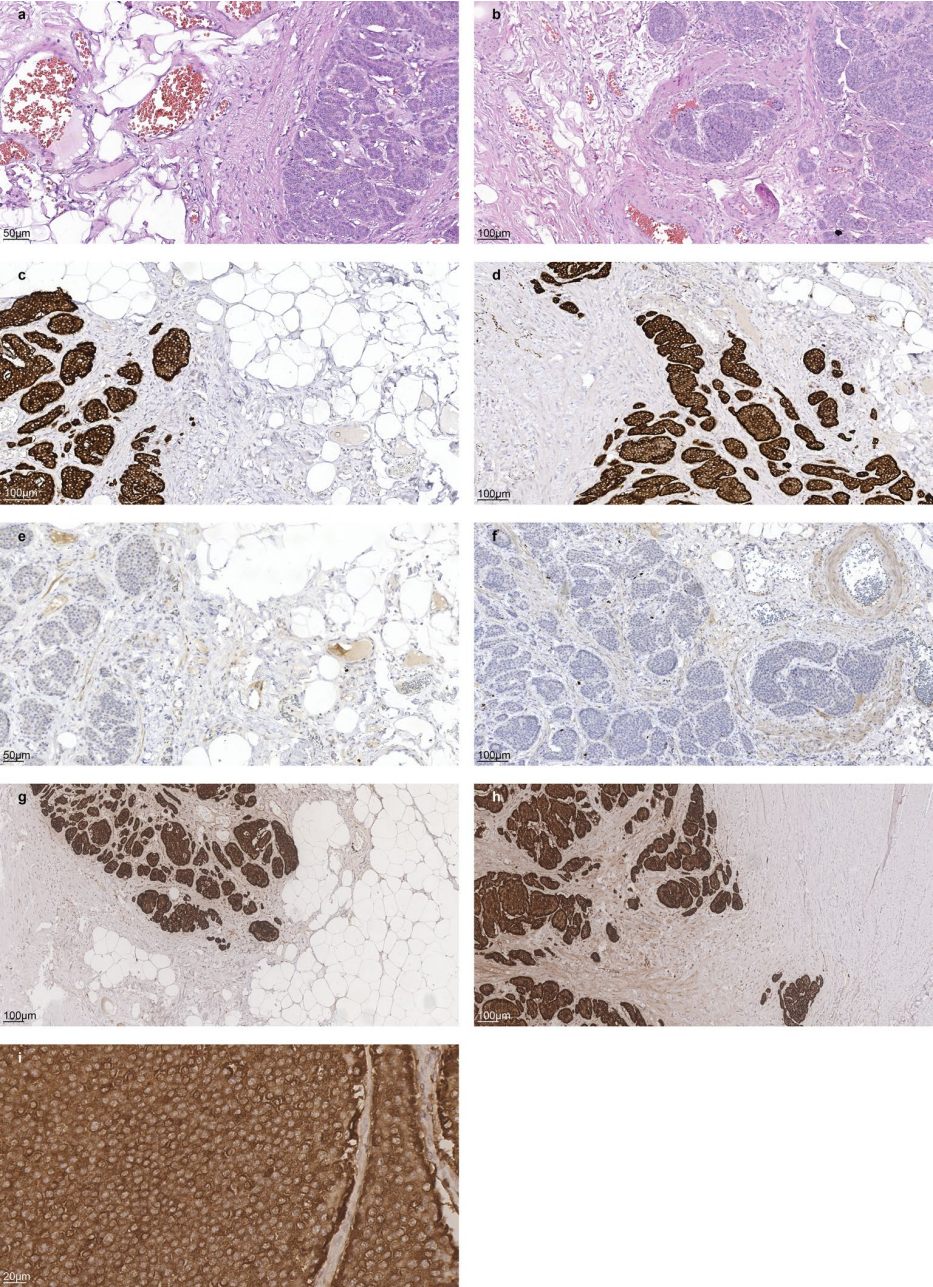
PanNET Histology and IHC. (a) Pancreatic tissue exhibiting tumor growth characterized by solid sheets and nests of rounded, relatively monomorphic cells. The periphery shows foci of lipomatosis and sclerosis, along with plethoric thin‐walled vessels. (b) Foci of vascular invasion. H&E stain, magnification ×200. (c, d) IHC reaction with Synaptophysin, an integral membrane glycoprotein localized in presynaptic neurosecretory vesicles (magnification ×200). Cytoplasmic expression is characteristic of well‐differentiated neuroendocrine tumors (carcinoid tumors) and neuroendocrine carcinomas. The pancreatic tumor demonstrates pronounced cytoplasmic expression within the neoplastic cells. (e, f) The proliferation index, based on the nuclear expression of Ki‐67 in the tumor cells, is less than 1%. (g) IHC reaction with antibodies against Chromogranin (magnification ×100), a granin protein located in the secretory vesicles of neurons and endocrine cells. The pancreatic neuroendocrine tumor exhibits pronounced cytoplasmic expression. (h) The pancreatic neuroendocrine tumor demonstrates pronounced cytoplasmic expression, appearing in some areas as clusters of fine granules (magnification ×400).

The patient underwent uncomplicated R0 pancreatoduodenectomy (Whipple procedure). Pathology confirmed a well‐differentiated neuroendocrine tumor (grade 1, Ki‐67 < 3%) of the pancreatic head with perivascular growth (stage IA) PanNET (Figure 3). The proximal pancreatic resection margin and common bile duct margin were negative for tumor growth (pT1N0). She remained recurrence‐free on scheduled surveillance for 12 months.

### 2.3 Diagnosis of Metachronous PDAC (2023)

Routine surveillance CECT revealed a new 17 × 16 mm hypovascular mass in the pancreatic tail, exhibiting peritumoral infiltration, and abutment of the splenic vein (Figure 2c–e). Completion pancreatectomy with splenectomy and segmental left colonic flexure resection was performed at Sechenov University Clinical Hospital. Final pathology confirmed pancreatic ductal adenocarcinoma (G2) with foci of lymphovascular invasion and perivascular growth. Metastasis was found in 1 of 10 lymph nodes (2 mm diameter). No tumor growth was found in the peripancreatic fat (pT2N1L1). The spleen and colon were adherent to the pancreas due to severe fibrosis and fibrinous exudate without direct tumor invasion. The levels of tumor markers were significantly elevated (CA 19‐9: 1700 U/mL, CEA: 6 ng/mL) (Table 1, Figure 1b). Restaging confirmed localized disease without distant metastases.

**TABLE 1:** Dynamics of tumor‐associated markers beginning in April 2022.

Marker	1 month	8 month later	10 month later	11 month later	15 month later	21 month later	25 month later
CEA (*N* < 5 ng/mL)	< 0.5 ng/mL	6 ng/mL	5 ng/mL	27 ng/mL	17 ng/mL	12 ng/mL	49 ng/mL
CA 19‐9 (*N* < 34 U/mL)	4.6 U/mL	1700 U/mL	800 U/mL	3125 U/mL	2565 U/mL	980 U/mL	1304 U/mL

### 2.4 Treatment of PDAC and Postoperative Course

**FIGURE 4:**
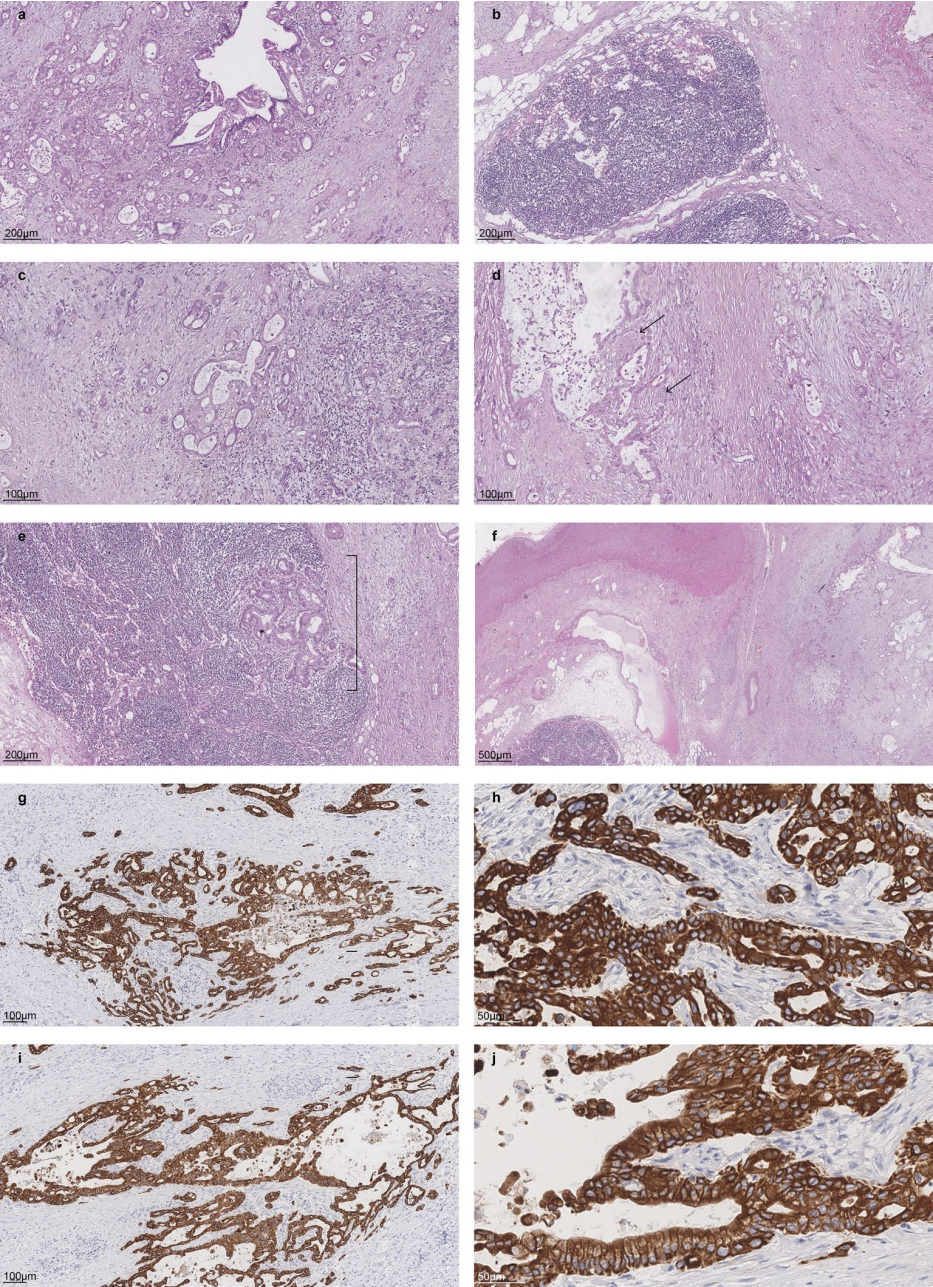
PDAC Histology and IHC. (a) Diffuse growth of ductal adenocarcinoma adjacent to a dilated, preserved duct. (b) Reserve lymph nodes showing sinus histiocytosis, the wall of a large vein, a solitary nerve trunk, and fibroadipose tissue. (c) Diffuse growth of ductal adenocarcinoma. Some tumor structures are embedded within fibrous tissue. (d) Foci of perineural invasion by ductal carcinoma. (e) Metastatic involvement of a lymph node. (f) Carcinoma growing within areas of fibrosis, an ectatic lymphatic vessel, and the wall of a large vein. Hematoxylin and eosin (H&E) staining. (g) IHC reaction with antibodies against Cytokeratin 7 (CK7)—a marker for non‐keratinizing epithelial cells—showing membranous and cytoplasmic expression. The micrograph illustrates positive membranous and cytoplasmic expression in the epithelial cells of the acinar, trabecular, and tubular tumor structures (magnification ×100). (h) Pronounced membranous and cytoplasmic expression in the epithelial cells of the acinar, trabecular, and tubular tumor structures (magnification ×400). (i) IHC reaction with antibodies against Cytokeratin 19 (CK19), which is frequently co‐expressed with CK7; expression is both membranous and cytoplasmic. The micrograph demonstrates positive membranous and cytoplasmic expression in the epithelial cells of the acinar, trabecular, and tubular tumor structures (magnification ×130). (j) Pronounced membranous and cytoplasmic expression in the epithelial cells of the acinar, trabecular, and tubular tumor structures (magnification ×400).

Completion pancreatectomy with splenectomy and segmental left colonic flexure resection (due to intraoperative suspicion of colonic involvement) was performed. Final pathology confirmed pT2N1M0 (stage IIB) PDAC (G2), with lymphovascular invasion, perineural invasion, and metastasis in 1 of 10 lymph nodes (Figure 4). Margins were R0; colonic and splenic involvement represented adhesion/fibrosis rather than direct invasion.

The immediate postoperative course was complicated by hyperglycemia (new‐onset insulin‐dependent type 3c diabetes mellitus) and reactive thrombocytosis. The patient's platelet count rose significantly after surgery (Figure 1d).

These complications, combined with significant nutritional deficiency, asthenia, and an Eastern Cooperative Oncology Group (ECOG) score of 3, precluded adjuvant chemotherapy. The levels of tumor markers decreased postoperatively (CA 19‐9: 800 U/mL, CEA: 5 ng/mL).

### 2.5 Disease Progression and First‐Line Therapy

Three months post‐pancreatectomy, surveillance CECT revealed new hypovascular liver lesions (segments II, III, IVb, V, VI, and VII) (Figure 2f,g). In addition, infiltration of the retroperitoneal fat was observed, as well as hypervascular and heterogeneous lymph nodes in the retroperitoneal, mesenteric, and portal regions, with short‐axis diameters of up to 10 mm. The levels of tumor markers increased sharply (CA 19‐9: 3125 U/mL, CEA: 27 ng/mL). Given her performance status (which improved to ECOG score of 1) and metastatic burden, first‐line palliative chemotherapy with gemcitabine monotherapy (1400 mg + 0.9% NaCl 250 mL, days 1, 8; q21 days) was initiated in December 2023.

### 2.6 Response to Gemcitabine

Imaging demonstrated a stable disease per RECIST 1.1 (a 15% decrease in the sum of the diameters of the hepatic target lesions was observed; stable lymphadenopathy) (Figure 1c). The levels of tumor markers decreased (CA 19‐9: 2565 U/mL, CEA: 17 ng/mL).

However, Grade 3 thrombocytopenia (platelets: 43 × 10^9^/L) occurred after Cycle 3, requiring platelet transfusion and omission of day 8 (Cycle 4). Platelets recovered by Cycle 5, allowing full dosing for Cycles 5–11.

After 11 cycles (7 months), the tumor marker levels decreased (CA 19–9: 980 U/mL, CEA: 12 ng/mL). Disease status remained stable.

### 2.7 Progression and Second‐Line Therapy

Imaging 3 months later revealed unequivocal progression in the liver (target lesions in S4b: 30 mm, S5: 33 mm; > 30% increase from nadir) (Table 2, Figure 2j,k). The levels of tumor markers increased (CA 19‐9: 1304 U/mL, CEA: 49 ng/mL). A retrospective review confirmed that progression was evident on prior scans but was missed due to interpretation variability outside the tertiary center (Figure 2h,i). Gemcitabine was discontinued. Second‐line therapy with irinotecan monotherapy (175 mg + 0.9% NaCl 500 mL, days 1, 8, 15; q28 days) was initiated in March 2025. The patient currently tolerates treatment well (ECOG 0–1), with restaging planned after Cycle 3.

**TABLE 2:** Dynamics of metastatic liver lesion size since 2023.

	1 month baseline	11 month later	15 month later	18 month later	22 month later	25 month later
S5	Absent	16 mm	14 mm	14 mm	27 mm	33 mm
S4b	Absent	17 mm	15 mm	14 mm	25 mm	30 mm

## 3 Discussion

Surgical treatment of pancreatic neoplasms predominates over pharmacological therapy because of the high chemoresistance of these tumors, particularly PDAC. When radical treatment is not feasible, systemic therapy largely determines patient prognosis [10].

Current guidelines from the American Society of Clinical Oncology (ASCO) and the National Comprehensive Cancer Network recommend different treatment regimens depending on the patient's overall condition [4, 5]. Patients with good performance status (ECOG 0 or 1) are generally offered palliative chemotherapy regimens, including folinic acid, fluorouracil, oxaliplatin, irinotecan [FOLFIRINOX], or gemcitabine combined with nab‐paclitaxel. In contrast, patients with poor performance status (ECOG score ≥ 2) typically receive gemcitabine monotherapy or best supportive care [4].

This clinical case must be interpreted within the paradigm of multiple primary neoplasms (MPNs). According to the classical criteria established by Warren and Gates, MPN is defined as the presence of two or more independent malignant tumors with distinct histological structures, provided that the possibility of one lesion being a metastasis of the other is excluded [11]. On the basis of the diagnostic time interval, the second tumor is classified as synchronous (detected simultaneously with the first or within 6 months) or metachronous (diagnosed after 6 months) [12]. Synchronous tumors, diagnosed within 6 months of each other, often share a common or simultaneous oncogenic driver, which frequently necessitates a unified, often palliative, treatment strategy. Recent literature on pancreaticobiliary collision tumors, such as the case of a PanNET and intra‐ampullary adenocarcinoma, emphasizes that such simultaneous presentations are exceptionally rare and often present a diagnostic dilemma [13]. In collision tumors, it is frequently challenging to distinguish between a mixed neuroendocrine‐nonneuroendocrine neoplasm and a true collision of independent primaries, requiring exhaustive histopathological analysis. From a management perspective, the distinction between synchronous and metachronous development is fundamental to prognosis. Synchronous or collision tumors often dictate a unified, frequently palliative strategy because the most aggressive component typically drives the prognosis, and the surgical window may be limited by simultaneous tumor burdens. Conversely, a metachronous presentation theoretically allows for sequential curative‐intent therapies tailored to each specific histology.

Our patient was initially diagnosed with a G2 PanNET, for which she underwent radical resection. The development of a new mass in the tail of the pancreas 12 months later, which was histologically verified as PDAC, fully satisfied the criteria for metachronous MPN. Genetic testing for germline mutations in the BRCA1 and BRCA2 genes yielded negative results; this excludes a link to hereditary cancer syndromes (such as BRCA‐associated syndromes) [14] and supports the sporadic nature of the disease. This finding fundamentally dictated the subsequent management strategy: unlike the potential recurrence or metastasis of the PanNET, the presence of a second primary malignancy necessitated a shift toward adjuvant chemotherapy with gemcitabine plus nab‐paclitaxel, the standard first‐line regimen for treating PDAC. The negative BRCA status also limits potential future targeted therapy options, underscoring the importance of classical chemotherapy regimens.

This clinical scenario raises critical questions regarding diagnosis and pathogenesis. Unlike synchronous tumors, metachronous tumors of distinct histology present a unique clinical and biological challenge. Their development following a significant disease‐free interval prompts consideration of the “field cancerization” phenomenon in the target organ, the long‐term effects of latent oncogenic exposures, and the high risk of misinterpreting the new tumor as a late metastasis of the initial disease [12, 15]. This differentiation is of fundamental importance for patient prognosis and therapeutic selection. Synchronous tumors often require a multimodal approach in which all components are targeted simultaneously, which could limit opportunities for radical treatment. Conversely, metachronous development—as seen in our case—theoretically allows for a sequential therapy that is maximally specific to each component, potentially improving the overall oncological outcome.

The rarity and value of the presented case are highlighted by its comparison with an observation described by Murokawa et al. [16], which involved multiple synchronous cancers of the pancreas and extrahepatic bile ducts. Although both cases illustrate the phenomenon of multiple primary tumors in the pancreatobiliary zone, their biology and clinical implications are diverse. Synchronous occurrence suggests a common or simultaneous carcinogenic driver and dictates the need for a unified, often palliative, strategy from the moment of diagnosis. In contrast, the metachronous development of PDAC following a cured PanNET points to either sequential independent oncogenic events or the prolonged manifestation of a genetic predisposition unrelated to the major known hereditary cancer genes (BRCA1/2). This 12‐month interval between histologically heterogeneous primary tumors is unique and demonstrates how successful treatment of the first tumor may “clear” the clinical field for the emergence of a second malignancy. This contrast reinforces the educational value of our report, emphasizing that the concept of “multiple primary tumors” encompasses fundamentally different clinical realities requiring distinct protocols for dynamic surveillance and treatment.

Despite the priority of systemic therapy in advanced disease, surgical resection remains the only curative treatment method for localized pancreatic tumors. The primary indications for pancreatectomy are the feasibility of achieving negative resection margins (R0) and the absence of vascular invasion or distant metastases. However, even when technically feasible, surgical outcomes depend critically on a constellation of factors: patient performance status (ECOG), tumor burden, and comorbidities.

Despite potential benefits, pancreatic surgery carries a high risk of postoperative complications, ranging from 20% to 50%. The most common complications are pancreatic fistula, hemorrhage, and wound infections; total pancreatectomy may also result in pancreatogenic diabetes mellitus [17, 18, 19]. To stratify this risk, the ACS NSQIP Surgical Risk Calculator was developed, which accounts for a complex of predictors: age, sex, body mass index (BMI), functional and anesthetic risk (ASA class), presence of cardiovascular pathology, dyspnea, coagulopathy, and the extent of the planned intervention [20]. During preparation for the first surgery (pancreaticoduodenectomy), our patient's risk profile (BMI 18.78, an ASA I–II class, and an absence of risk factors such as obesity, sepsis, or dyspnea) was assessed as “below‐average” which was consistent with her successful postoperative recovery. This example illustrates the importance of thorough preoperative evaluation and mandatory patient counseling regarding potential risks and expected outcomes, even in patients with a formally favorable prognosis [21, 22].

Thus, the presented case of metachronous PanNET and PDAC serves as a crucial illustration of the complexities involved in differential diagnosis, the necessity for prolonged oncological vigilance in patients following radical treatment, and the importance of developing a personalized therapeutic strategy on the basis of precise histological verification of each tumor component. The negative BRCA1/2 test result in this patient highlights that, even in the absence of obvious hereditary syndromes, the risk of developing sequential primary tumors in the pancreatobiliary zone remains significant. This case underscores the need for further research to identify other genetic and epigenetic markers that could assist in risk stratification and the early detection of such rare but clinically aggressive scenarios.

A critical educational aspect of this case is the retrospective identification of disease progression that was initially overlooked during routine monitoring outside of our tertiary center. In November 2023, hypovascular hepatic lesions were identified in segments IVb and V, measuring 17 and 16 mm, respectively. While subsequent imaging in October 2024 showed these lesions had grown to 25 mm and 27 mm, this significant interval growth was not immediately flagged as progression. This underscores the inherent challenges in radiological assessment for patients with rare, sequential malignancies like PanNET and PDAC. It serves as a vital teaching point: specialized oncological centers provide the multidisciplinary expertise and consistent longitudinal comparison necessary to detect subtle changes in tumor burden. Ensuring that surveillance imaging is reviewed by radiologists experienced in pancreatobiliary oncology is essential to prevent therapeutic delays and optimize the timing of second‐line interventions.

## 4 Conclusion

The presented clinical case of metachronous multiple primary pancreatic neoplasms (PanNET followed by PDAC) exemplifies a critical challenge in modern oncology: the complex interplay between rare tumor biology and clinical management. The primary barriers to implementing a personalized approach remain significant intra‐ and intertumoral heterogeneity, which limits the availability of actionable therapeutic targets [23, 24], difficulties in obtaining and timely sequencing of high‐quality biopsy material for molecular profiling, and rapid clinical deterioration, which frequently restricts therapeutic options [24].

However, a key conclusion is that these barriers are surmountable. Achieving a meaningful period of disease control—specifically, 7.5 months without progression on first‐line therapy—within such an aggressive clinical scenario was the result of a consistent, histology‐driven strategy, integral to the systematic efforts of a multidisciplinary team [25, 26, 27]. Cohesive collaboration among the team ensured the correct interpretation of the metachronous process by ruling out metastatic disease, facilitated the timely transition to appropriate chemotherapy, and provided ongoing dynamic patient monitoring.

Drawing on this experience, two interconnected avenues for optimizing the management of such complex cases can be identified. The first is the implementation of a dynamic tumor re‐evaluation protocol via the standardization of mandatory morphological repeats and, where feasible, molecular assessment upon the occurrence of any new tumor event. The second is the integration of technologies to monitor tumor evolution, including the routine incorporation of dynamic molecular screening such as liquid biopsy [28, 29] and ensuring broad access to next‐generation sequencing [30] to identify new driver alterations and adapt therapy in real time.

Thus, this case demonstrates that the synergy between a multidisciplinary approach and emerging diagnostic technologies is a prerequisite for the transition to truly personalized therapy and for improving outcomes in patients with the most complex forms of pancreatic cancer.

## Author Contributions

**Dina F. Islamova:** conceptualization, investigation, writing – original draft, formal analysis. **Pavel Burko:** investigation, visualization, writing – review and editing, formal analysis, data curation. **Anastasia S. Fatyanova:** conceptualization, investigation, writing – original draft, methodology, writing – review and editing, supervision. **Ilias Miltiadis:** writing – original draft, writing – review and editing, conceptualization, methodology, formal analysis, visualization, investigation. **Julia V. Lerner:** conceptualization, investigation, writing – original draft, writing – review and editing. **Maria E. Kalinina:** conceptualization, investigation, writing – original draft, writing – review and editing, formal analysis, data curation. **Igor O. Shchekoturov:** conceptualization, investigation, writing – original draft, formal analysis, software. **Nadezhda A. Stashevskaya:** conceptualization, formal analysis.

## Funding

The authors have nothing to report.

## Ethics Statement

Institutional review board approval was not required for this retrospective case report in accordance with the national guidelines of the Russian Federation. According to Ministry of Health Order No. 200n and Federal Law No. 61‐FZ, mandatory ethical review is strictly required only for prospective clinical trials involving experimental interventions. As this report details standard clinical practice, it is exempt from institutional review board approval.

## Consent

Written informed consent was obtained from the patient for publication of this case report and accompanying images.

## Conflicts of Interest

The authors declare no conflicts of interest.

## Data Availability

The data that support the findings of this study are available from the corresponding author upon reasonable request.
